# Quality research in healthcare: are researchers getting enough statistical support?

**DOI:** 10.1186/1472-6963-6-2

**Published:** 2006-01-12

**Authors:** Rumana Z Omar, Nick McNally, Gareth Ambler, Allyson M Pollock

**Affiliations:** 1 Research and Development, University College London Hospitals NHS Foundation Trust, Maple House, 149 Tottenham Court Road, London W1P 9LL, UK; 2 Department of Statistical Science, University College London, Gower Street, London WC1E 6BT, UK; 3 School of Public Policy, University College London, Gower Street, London WC1E 6BT, UK

## Abstract

**Background:**

Reviews of peer-reviewed health studies have highlighted problems with their methodological quality. As published health studies form the basis of many clinical decisions including evaluation and provisions of health services, this has scientific and ethical implications. The lack of involvement of methodologists (defined as statisticians or quantitative epidemiologists) has been suggested as one key reason for this problem and this has been linked to the lack of access to methodologists. This issue was highlighted several years ago and it was suggested that more investments were needed from health care organisations and Universities to alleviate this problem.

**Methods:**

To assess the current level of methodological support available for health researchers in England, we surveyed the 25 National Health Services Trusts in England, that are the major recipients of the Department of Health's research and development (R&D) support funding.

**Results and discussion:**

The survey shows that the earmarking of resources to provide appropriate methodological support to health researchers in these organisations is not widespread. Neither the level of R&D support funding received nor the volume of research undertaken by these organisations showed any association with the amount they spent in providing a central resource for methodological support for their researchers.

**Conclusion:**

The promotion and delivery of high quality health research requires that organisations hosting health research and their academic partners put in place funding and systems to provide appropriate methodological support to ensure valid research findings. If resources are limited, health researchers may have to rely on short courses and/or a limited number of advisory sessions which may not always produce satisfactory results.

## Background

Published health studies form the basis of many clinical decisions including the evaluation and provision of health services. However, reviews of peer reviewed literature show that the scientific quality of such studies is often compromised by the use of inappropriate methodology [1-5]. This has scientific and ethical implications in health care. Although a majority of the health studies are quantitative in nature, it has been shown that a large proportion of these do not involve methodologists (defined as someone with at least a postgraduate degree in Statistics, or in Epidemiology with substantive statistical components) [6,7]. This lack of involvement may be partly due to inadequate access to methodologists [7]. It was noted more than a decade ago that the practice of health researchers performing their own statistical analysis without appropriate training is not satisfactory, regarding both the accuracy of the results produced and the use of their time [7]. At that time it was suggested that healthcare organisations and Universities should invest more to provide good quality methodological support for health researchers to alleviate this problem [7].

Recently in England the Department of Health (DH) has published a research governance (RG) framework which defines responsibilities and sets scientific, ethical and legal standards for health research [8,9]. Central to the implementation of the RG framework in the National Health Services (NHS) in England are Hospital and Primary Care Trusts because of their role as sponsors, hosts and employers. Currently over 400 million pounds is spent annually by the DH supporting Research and Development (R&D) activity across the NHS. This funding is intended to meet the costs which NHS Providers incur by hosting, undertaking or commissioning R&D. A total of 261 organisations including Hospital and Primary Care Trusts received R&D Support Funding in 2003/04. Despite the very large volume of research carried out by these organisations [10], no formal evaluation has been done to assess whether the level of investment and methodological support provided by the Trusts was adequate for their researchers. In this paper we present the results of a survey regarding the provision of methodological support available to health researchers by the NHS Trusts' R&D Departments in England. We also discuss how an approach may be adopted to provide researchers with the necessary support.

## Methods

In January 2004 we conducted a survey in of the 25 major recipients of NHS R&D support funding in England (constituting more than 80% of the total R&D support funding allocation), regarding their provision of methodological support for their health researchers. We identified these 25 Trusts from the DH's website [11]. A questionnaire was designed jointly by statisticians, public health researchers and an R&D Manager for this survey. It was piloted in 2 Trusts and amended to clarify any ambiguity in the questions. The amended questionnaire was sent to the Trusts' R&D managers asking about:

• Provision of methodological support

• Level of dedicated funding for methodologists (defined as before). This was categorised as <£10,000, £10,000–£40,000 and >£40,000, to reflect the funding appropriate for, respectively, 1 day a week support for a junior post, a full time junior or a part time senior post, and at least 1 full time senior or more than 1 part/full time junior posts.

• Alternative arrangements if no dedicated funding was available

• Level of methodological support provided free of charge

We viewed the Trusts' R&D websites for further information. We consulted the National Research Register [10] to obtain information on the number of research projects registered by the R&D Departments of these 25 Trusts.

We investigated the level of dedicated funding for methodological support provided by Trusts' R&D departments in relation to their level of allocated DH R&D support funding and the number of research projects registered using Spearman's rank correlation. We show these relationships graphically by ranking the Trusts by their funding allocations/number of projects, then splitting them into 3 tertile groups and producing bar charts of dedicated funding.

## Results

**Figure 1:**
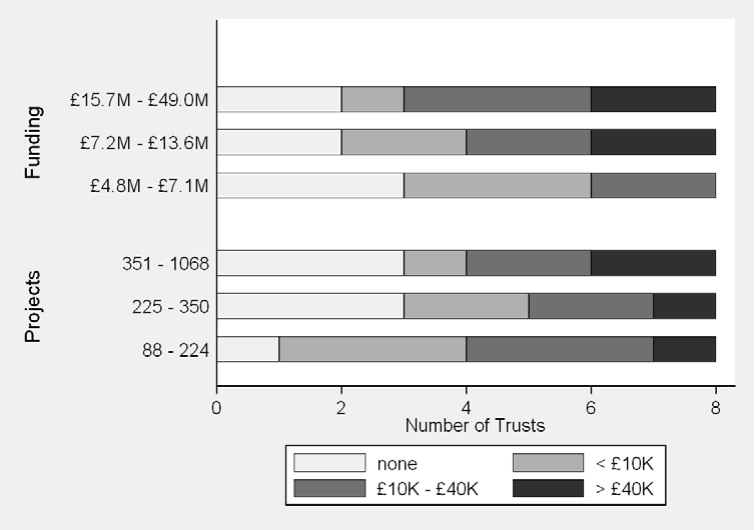
NHS Trust Research and Development spending in 2003–2004 on methodological support by funding allocation and number of registered Research Projects.

All 25 Trusts responded. We do not present the results for individual Trusts so as to preserve their anonymity. The median funding allocated to these Trusts in 2003–04 was £8.2 millions (range: £4.8 – £49 millions) [11]. The median number of research projects registered by these Trusts with the National Research Register in 2003–04 was 237 (range: 88 – 1068) [10]. Eighteen Trusts reported to have a dedicated funding for a methodologist. This included one consortium of 4 Trusts where funding was available in only 1 of these 4 Trusts. Only 4 had a budget exceeding £40,000. However, one of these 18 Trusts did not report the level of R&D funding allocated for methodological support. Of the 7 Trusts with no dedicated funding, 4 reported to have an arrangement with a local University or another partner and the remaining 2 did not appear to offer any form of support. For 2 Trusts the only support that was available was short statistical advisory sessions or advice via email from a statistician. Fourteen Trusts offered free methodological advice to all Trust researchers, although this was limited to a 1 hour session in some of these Trusts. Within the 24 Trusts that provided complete information, there did not appear to be any relationship between the R&D funding for methodological support and the annual number of registered projects (figure 1, spearman rank correlation -0.01). There was only a weak relationship if any with the NHS R&D support funding allocation (figure 1, spearman rank correlation 0.33).

## Discussion

Our survey shows that the practice of earmarking resources to develop a central system for appropriate methodological support is not widespread in the NHS Trusts in England Although this problem in health care organisations was highlighted more than a decade ago, little seems to have changed since then in England.

If resources are limited, health researchers may have to perform their own analysis, relying only on short courses or a limited number of advisory sessions. Although these arrangements are better than having no methodological support at all, they may not always produce satisfactory results.

Ideally all health care organisations hosting research should develop a central resource for methodological support, for example a Medical Statistics/Biostatistics Unit. This should be formed in collaboration with their academic partners in order to attract high quality methodologists with their own research programmes. This should enable support from experienced methodologists or supervised support from the junior ones. It will also facilitate specialist support if needed. The number of methodologists should be appropriate for the volume of quantitative research carried in the organisation. Unlimited support free of charge should be provided at the initial stages of a study: study design and preparation of grant applications/protocols. A methodologist's involvement in data analysis should be funded by research grants where available or possibly subsidised by the R&D or a similar department, depending on the quality of the research and the research priorities of the organisation. For the data analysis to be done effectively, the methodologist should be an integral part of the research team. Limited free support could be provided for supervising data analysis if the researchers sought advice at the study design stage and the analytical requirement is simple. Ideally the waiting time to see a methodologist should not be too long, possibly not exceeding a couple of weeks. Additionally, the methodologists should contribute to the organisation's research governance activities through training programmes, methodological audits and the development of methodological guidelines. Audits of research protocols, statistical needs etc. could be used as means to target support to researchers. Our own Trust is working towards developing a central resource of methodological support similar to the one described above.

However, having a structured system for methodological support in organisations which host health research may not always be sufficient to ensure high methodological quality of studies. Organisations should also adopt strategies to ensure that the service is being used effectively by their researchers. This could be done by raising awareness among their researchers to recognise the need for support and to monitor whether research projects have appropriately involved methodologists where necessary. For example, the RG framework in England requires that all research in NHS Trusts should have an independent expert review either through the peer review process of grant bodies or an internal review process within the Trusts [9]. These review processes should ensure that a research team has the necessary expertise to conduct their proposed research to a high standard. The Royal Statistical Society recommends that a statistical review should be provided for research projects during the approval stage by the R&D department of each Trust [12]. At the same time the researchers should assess when and to what extent their projects need methodological support and to budget for it appropriately when applying for research grants.

Our survey is focussed on support available from NHS Trust's R&D Departments or via their links with other supporting bodies. This maybe seen as a limitation as some health researchers may work in departments which employ their own methodologists. However, this does not address the problem for those researchers who do not have this facility. Furthermore, if a junior methodologist is working in isolation within these departments the level and quality of support may not be always be adequate [7].

Reviews of published literature in health journals show that the use of poor methodology in health research is not confined to England only (1–5). In the USA, following calls from the Institute of Medicine of the National Academies of Science for the overhaul of research approval processes, many institutions are implementing systems of research oversight that separate out procedures for scientific review from the ethics review process [13].

## Conclusion

Our survey has shown that the practice of earmarking resources to develop a central system for appropriate methodological support is not widespread in the NHS Trusts in England. The promotion and delivery of high quality health research requires that organisations hosting health research and their academic partners put in place funding and systems to provide sufficient methodological support to ensure valid research findings. It is also important to evaluate how the development of a high quality central resource for methodological support has affected the scientific quality of research in organisations that had adopted such a strategy. This would aid in the planning and targeting of organisational resources for research support.

## Abbreviations

DH: Department of Health

NHS: National Health Services

R&D: Research and Development

RG: Research Governance

USA: United States of America

## Competing interests

The author(s) declare that they have no competing interests.

## Authors' contributions

RO and AP conceived the idea for the paper.

RO, NM, GA and AP designed the questionnaire.

RO and GA analysed the data.

RO, NM, GA, AP contributed to the writing of the paper.

## Pre-publication history

The pre-publication history for this paper can be accessed here:
